# Foamy Cell Angiosarcoma in a Patient with Xeroderma Pigmentosum: A Case Report and Comprehensive Review of the Literature

**DOI:** 10.30699/IJP.2022.539239.2729

**Published:** 2022-08-13

**Authors:** Fatemeh Montazer, Ali Zare Dehnavi, Abbas Dehghani, Arash Maboudi, Azadeh Goodarzi

**Affiliations:** 1 *Department of Pathology, Firoozabadi Hospital, Iran University of Medical Sciences, Tehran, Iran*; 2 *Tehran University of Medical Sciences, Tehran, Iran*; 3 *Department of Dermatology, Rasool Akram Medical Complex, School of Medicine* *، * *Iran University of Medical Sciences (IUMS), Tehran, Iran*; 4 *Private Practitioner, Dermatologist, Amol, Mazandaran, Iran*; 5 *Department of Dermatology, Rasool Akram Medical Complex Clinical Research Development Center (RCRDC), School of Medicine, Iran University of Medical Sciences, Tehran, Iran*

**Keywords:** Angiosarcoma, Histopathology, Foamy cell, Malignancy, Skin cancer, Xerodermapigmentosum

## Abstract

Xeroderma pigmentosum (XP) is a rare autosomal recessive disorder characterized by a DNA repair defect caused by ultraviolet light and cutaneous manifestations, including solar lentigines, xerosis, actinic damage, and cutaneous neoplasms (e.g., basal cell carcinoma, squamous cell carcinoma, and melanoma). Cutaneous angiosarcoma (AS) is a rare group of aggressive skin tumors that infrequently occur in patients with XP, usually involving the scalp or face. The AS has three subtypes: idiopathic, complicating lymphedema, and post-irradiation. The AS has diverse histopathological types, and the uncommon variants are clear cell, epithelioid, granular cell, pseudo lymphomatous, verrucous, and signet-ring cell variants. Although the foamy cell variant of AS is the rarest type, its diagnosis would be really challenging due to the wide variety of differential diagnoses, especially for poorly differentiated ones. Therefore, definitive diagnosis and effective management in the early stages are crucial, and immunohistochemical (IHC) tests are essential. Here we report a 50-year-old Iranian man with AS complicating XP who presented with an ulcerative erythematous and progressive plaque. Histopathologic studies revealed foamy cells and vascular markers (i.e., CD 31 and CD 34) were positive, immunohistochemically which was found unusual features. In addition,, we review previously reported cases in the literature to provide some information on the diagnosis and management of such cases.

## Introduction

Xeroderma pigmentosum (XP) is a rare inherited condition transmitted in an autosomal recessive pattern (1-3). The main problem in more than 80% of cases is nucleotide excision repair (NER) defect causing a disturbed function of DNA repair harmed by sunlight ultraviolet radiation (1,4). This heightened photosensitivity results in sunburn, pigmentary changes, accelerated skin aging, anda significantly elevated incidence of skin neoplasms, including basal cell carcinoma, squamous cell carcinoma, and melanoma (5, 6). Angiosarcomas (ASs) are a group of rare cutaneous and soft tissue sarcomas with a high tumor-related mortality rate that infrequently arise in XP cases (7-10).

Clinically, ASs are categorized into three distinctive settings: 1. Idiopathic type, classically on the head and neck of elderly patients, 2. Stewart-Treves type on lymph edematous limbs, and 3. Post-radiation type on the site of prior irradiation (11, 12). Histopathologically, ASs have a wide range of appearances, even in different sections of the same lesion (11). They are microscopically heterogeneous due to the degree of differentiation (13). Well-differentiated lesions consist of well-formed vascular spaces, while poorly differentiated ones appear with epithelioid or spindle cell patterns (9, 11, 14). The latter group is a real diagnostic challenge due to the wide range of differential diagnoses, including melanoma, lymphoma, and atypical fibroxanthoma. Consequently, variable immunohistochemical (IHC) studies are essential for definite diagnosis (15). Several rare variants of AS resulting in a signet ring or foamy cell appearance have been reported in a few reports that mimic other neoplasms and are a substantial diagnostic challengechallenging histopathologic diagnosis, diversity of variants, poor prognosis, and aggressive nature of AS (17, 18). Here, we present an XP case with angiosarcoma (AS) and foamy cells in the histopathologic examination. Furthermore, we review the findings of all the previously reported cases in the literature to provide more information on their diagnosis and management.

## Case Presentation

**Fig 1:**
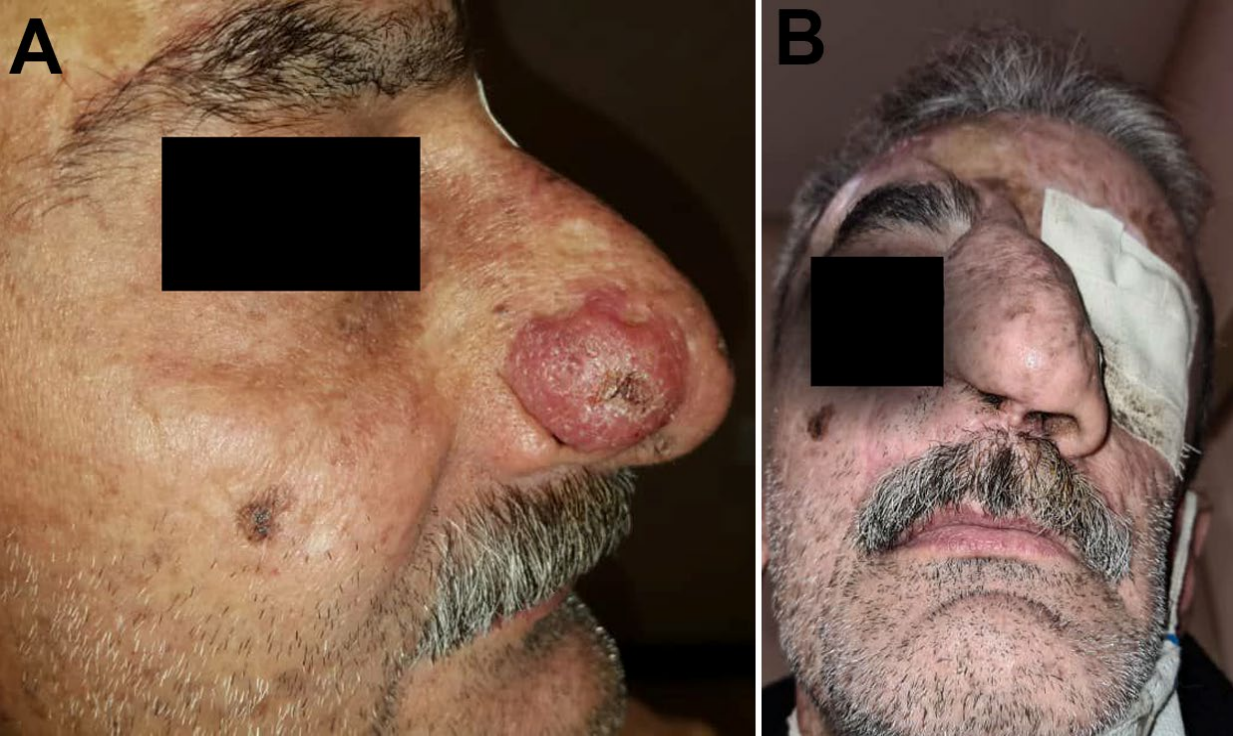
(A, B), Clinical features. A: a 1.6 × 1.3 cm sized ulcerative erythematous, non-bleeding nodule on the right Ala. B: After the excision of the lesion and nasal tissue reconstruction with the forehead flap

 The case is a known case of XP diagnosed since the age of 25 years with history of several recurrent BCCs and SCCs on sun-exposed areas (i.e., face, ear, and orbit) for 30 years. The patient had undergone several surgical excisions for those lesions. The patient had been born to non-consanguineous parents with no family history of XP. He presented to our clinic at age of 50 with the complaint of a progressive lesion on his nose of 2 years duration. Physical examination showed a 1.6×1.3 cm ulcerative erythematous non-bleeding progressive plaque on the right ala (Figure1). Further investigation showed no neurologic and ophthalmologic symptoms.

**Fig 2:**
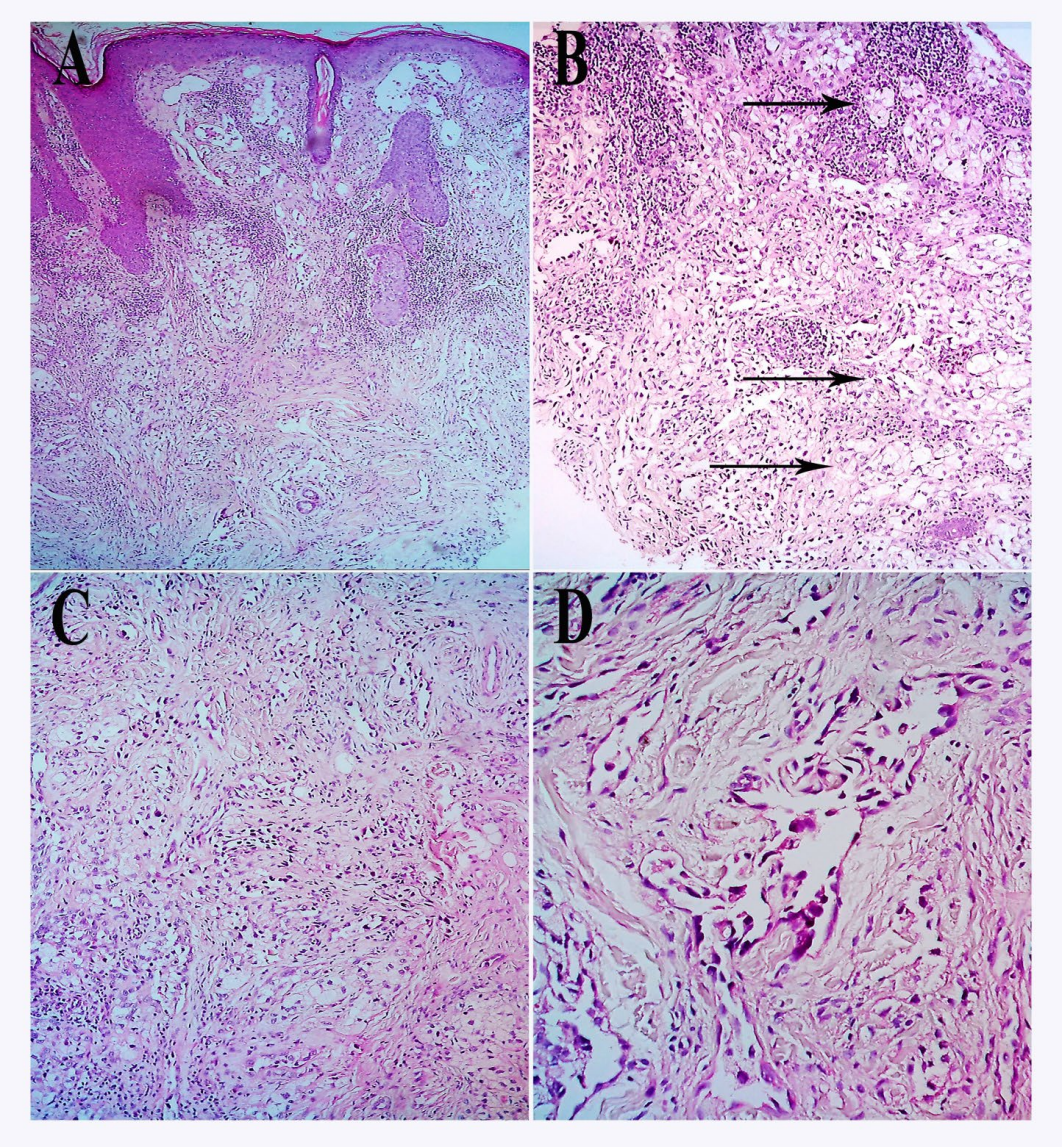
(A-D), H&E slides. A: Irregularly shaped anastomosing vascular channels (x100).B: Extensive foamy cell alteration of the tumoral cells(arrows) resembling a reactive xanthogranulomatous process (x200).C: Highly infiltrative architecture and poor demarcation (x200). D: Multilayering of endothelial cells, nuclear atypia & increased mitoses (x400)

**Fig 3:**
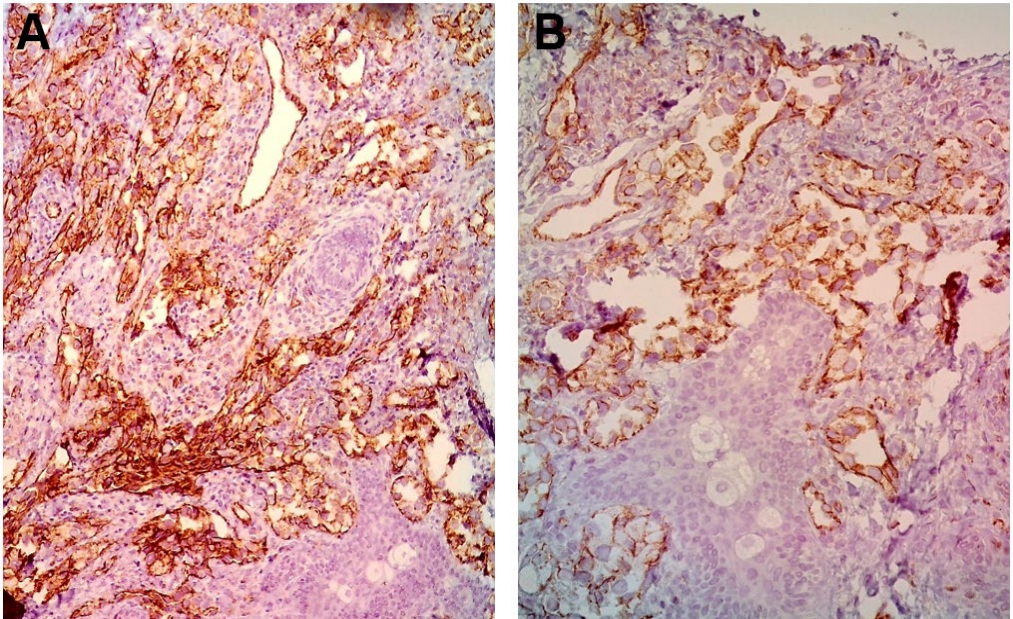
(A,B), immunohistochemical studies: Endothelial cell markers (x200) of CD31 (A) and CD34 (B) are strongly positive

A biopsy of the lesion was performed by surgical excision. Grossly, the specimen was hemorrhagic and necrotic. On microscopic appearance, numerous irregularly shaped anastomosing vascular channels with a highly infiltrative architecture and poor demarcation, which were lined by atypical endothelial cells, were present in the dermal component. In addition, dissecting collagen bundles were noted. Tumor cells were typically plump, hyperchromatic, pleomorphic, and mitotically active. Moreover, intratumoral foamy cells were frequently observed, and mild stromal lymphocytic aggregates were also present (Figure 2A-D). Immunohistochemicalmost tumoral cells expressed CD31 and CD34 with moderate intensity (Figure 3), and Ki67 was positive in about 10% of tumoral cells. They were negative for Pan CK, Melan A, S100, HMB45, CK7, CEA, and CD68.

According to the combination of histopathologic findings and immunohistochemical results, AS with foamy change was confirmed. The differential diagnoses included a reactive xanthogranulomatous process due to prominent foamy cell alteration of tumoral cells and clear cell dermatofibroma. Finally, the patient he underwent lesion excision with nasal tissue reconstruction with the forehead flap. The patient had no signs of recurrence or metastasis during 2-year of follow-up.

## Discussion

In contrast to classical skin tumors in XP patients, cutaneous AS is a group of infrequent malignant lesions. Heterogeneity among their histopathologic findings makes them more difficult to diagnose. To our knowledge, several cases of AS in XP patients and a few cases of AS with foamy cell appearance in the histopathologic studies have been reported (Tables 1 and 2)(17). However, no cases with foamy cell manifestation of an AS tumor in an XP patient have been described yet. We presented the first case of AS in an XP patient with rare microscopic findings due to the presence of foamy cells. 

**Table 1:** Clinical, histopathologic, immunohistochemical, and genetic findings and outcome of the previously reported cases of xeroderma pigmentosum and associated angiosarcoma

Variables	Present study	Won Jin Hong Study *(1)*	Matthew T. Olson Study *(8)*		J. LEAKE Study *(20)*
Gender	Male	Male	Female	Male	**Female**
Age at diagnosis (years)	50	65	11	73	**15**
Site of the Lesion	Ala of nose	Scalp	Oral cavity	Auricle	**Scalp**
Lesion characteristics	Erythematous ulcerated plaque	Two erythematous firm nodules	Fungating polypoid mass	Erythematous ulcerated plaque	**Rapidly growing nodule**
Complaints	Gradual growth	Progressive skin pigmentation, multiple lentiginous, xerosis	Recurrent lesion of the tongue	Bleeding lesion on the left auricle	**Rapidly growing nodule**
Ocular symptoms	No	NA	Cataracts (complete visual loss in the right eye and partial vision loss in the left eye)	NA	**NA**
Neurological symptoms	No	No	No	NA	**NA**
Past dermatologic history	Recurrent BCC, and SCC	BCC, Actinic keratosis	BCC, Recurrent lesion of the tongue	BCC, SCC Melanoma	**BCC, SCC**
Family history of XP	No	No	NA	Yes (sibling)	**NA**
Parents’ consanguinity	No	No	NA	NA	**No**
Histopathologic findings	Irregularly shaped anastomosing vascular channels lined by atypical endothelial cells with a highly infiltrative architecture and poor demarcation Intratumoral foamy cell changes	A proliferation of atypical endothelial cells with a network of anastomosing vessels, small vascular channels dissecting the collagen fibers	Rounded epithelioid cells with abundant eosinophilic cytoplasm, large vesicular nuclei, and prominent nucleoli. irregular anastomosing channels	Atypical endothelial cells with markedly hyperchromatic pleomorphic nuclei in the dermis, forming an undifferentiated vascular structure	**Infiltrative** **tumor, poorly differentiated, foci of plump endothelial cells lined** **anastomosing vascular channels, pleomorphic epithelioid and spindle-shaped cells with large nucleoli, phagocytosed hemosiderin pigment**
Immunohistochemical stains panel	CD31: Positive CD34: Positive Ki67: Positive (10%) PanCK: Negative Melan A: Negative S100, CK7, CEA, CD68: Negative	CD31: Positive HMB45: Negative HHV8: Negative	CD31: Positive CD34: Positive HHV8: Negative AE1/AE3: Negative P63: Negative	CD31: Positive CD34: Positive D2–40: Positive	**Von Willebrand: +** **Ulexewropaeus** **agglutinin type 1: +** **JC 70: +** **QBEND: +**
Gene	NA	POLH	NA	POLH	**NA**
Variant	NA	c.490G>T	NA	c.1066C>T	**NA**
Metastasis	No	No	NA	No	**NA**
Treatment	Excision with graft	Radiotherapy + excision	Radiotherapy + excision	Excision	**Excision**
Outcome	No recurrence for 2 years of follow-up	No recurrence for 4.5 years of follow-up	NA	No recurrence for 15 months of follow-up	**Recurred locally within 5 weeks**
Variables	Shilpi Sharma Study *(7)*	D.Ludolph-hauser Study *(21)*	Karkouche Study *(22)*	Karkouche Study *(22)*	**Karkouche** **Study ** ***(22)***
Gender	Male	Female	Male	Female	**Female**
Age at diagnosis(years)	25	13	18	21	**27**
Lesion site	Scalp	Shin	Left sub palpebral	Right internal canthus	**Parotid gland**
Lesion characteristics	Non-healing bleeding ulcer with irregular, rolled-out edges	NA	NA	NA	**NA**
Complaints	Non-healing bleeding ulcer	A lesion on the right shin	A lesion on sub palpebral	A lesion on the internal canthus	
Ocular symptoms	No	NA	NA	NA	**NA**
Neurological symptoms	No	NA	NA	NA	**NA**
Past dermatologic history	SCC, Myoepithelial carcinoma of the dermis, benign skin adnexal tumor of hair follicle differentiation	SCC, BCC, Actinic keratosis, Hemangiomas	BCC, actinic keratosis	BCC	**NA**
Family history of XP	Yes (2nd- and 3rd-degree relatives)	NA	NA	NA	**NA**
Parents’ consanguinity	No	First cousin	NA	NA	**NA**
Histopathologic findings	Disordered proliferation of atypical endothelial cells with hyperchromatic markedly pleomorphic nuclei and eosinophilic cytoplasm. Small vascular channels with red blood cells were seen lined by similar cells dissecting through the dermis and around the adnexal structure	Deep infiltrate of pleomorphous tumor cells	A vascular proliferation, with a network of anastomosing vessels lined by atypical endothelial cells	NA	**NA**
Immunohistochemical stains panel	CD31: Positive CD34: Positive Cytokeratin: Negative	CD31: Positive Factor VIII: Positive	CD31: Positive CD34: Positive	NA	**NA**
Gene	NA	NA	NA	NA	**NA**
Variant	NA	NA	NA	NA	**NA**
Metastasis	No	NA	NA	NA	**NA**
Treatment	Radiotherapy	Excision	Excision	Incomplete excision	**Complete resection**
Outcome	NA	NA	No sign of recurrence after 19 months	Recurred after 11 months	**No sign of recurrence after 27 months**

**Table 2:** Clinical, histopathology, and IHC findings of the previously reported cases of cutaneous foamy cell angiosarcoma

Case number	Author	Age	Gender	Location	Clinical findings	Histopathology	Immunohistochemistry
1	Ackerman* et al. *(23)	NA	NA	NA	NA	Focal areas of neoplastic cells with a foamy appearance	**NA**
2	Tatsas* et al. *(24)	73	M	Forehead	A Purpuric growing macule with a nodule	Dermal, diffuse involvement by large, pale, relatively monomorphous mononuclear cells with abundant vacuolated cytoplasm	**Positivity for CD31, CD34, Fli-1,** **Factor VIII-related antigen and** **podoplanin**
3	Tatsas* et al.*(24)	23	M	Shoulder	A nodule with color change	Dermal, diffuse involvement by large, pale, relatively monomorphous mononuclear cells with abundant vacuolated cytoplasm	**Positivity for CD31, CD34, Fli-1,** **Factor VIII-related antigen and** **podoplanin**
4	Svajdler* et al.*(11)	86	M	Scalp	An ulcerated plaque	Epithelioid cells with foamy cytoplasm and round, oval angulated nuclei indented by intracytoplasmic vacuoles with few mitoses.	**Positivity for vimentin, CD34,** **CD31, D2-40, Fli-1 and ERG and** **negativity for CD68, CD163,** **cytokeratin, EMA, CD10, S100,** **HMB45 and Melan A**
5	Wood* et al.*(15)	68	M	Face	A large plaque	Sheet-like growth of finely multivacuolated cells with centrally placed nuclei with no signs of indentation	**Positivity for CD31, ERG, factor** **VIII, CD68 and CD163 (finely** **granular cytoplasmic). Negativity for SMA, CD34, desmin, adipophilin, EMA, and cytokeratin.**
6	Wood* et al.*(15)	78	M	Scalp, forehead and face	Multinodular plaque	Sheet-like growth of finely multivacuolated cells with centrally placed nuclei with no signs of indentation	**Positivity for CD31, ERG, CD68, and CD163 (finely granular** **cytoplasmic). Negativity for S100protein, SMA, desmin, adipophilin, EMA, and cytokeratin.**
7	Llamas-Velasco* et al.*(17)	85	M	Scalp	A large plaque	Dermal diffuse infiltration of large cells with foamy cytoplasm intermingled with lymphocytes and erythrocytes.	**Positivity for CD31, CD34, ERG,** **podoplanin, Lyve-1, and NKIC3.** **Negativity for CD68 andlysozyme. Ser10 showed 3–4** **mitoses per high power field**
8	Curent case	50	M	Ala of nose	Erythematous ulcerated plaque	irregularly shaped anastomosing vascular channels lined by atypical endothelial cells with a highly infiltrative architecture and poor demarcation, Tumor cells were typically plump, hyperchromatic, pleomorphic, and mitotically active, and Intratumoral foamy cell changes were frequently seen, Mild Stromal lymphocytic aggregates	**Positive for CD31, CD34, KI67,** **Negative for PanCK, MelanA, S100, HBM45, CK7, CEA, and CD68.**

Co-occurrence of XP and AS is observed almost equally in both genders (Table 1). In contrast to our study and two other investigations (1, 19), most previously reported cases of AS occurred in XP patients, were younger adults. Approximately half of AS cases occurs on the head and neck skin (19). Regarding the site of lesion, almost all previously reported AS cases in XP patients have been located on the head and neck with a preference for the scalp with an exception on the shin (1, 21). Our patient's lesion was on the nose, which was the same as a previously described 15-year-old male case (1). Moreover, its size was <5 cm, similar to most prior cases (19).

The main clinical presentation of AS in XP patients is a progressive and likely ulcerative erythematous plaque or nodule. Recurrent BCCs and SCCs had occurred in this case for a long time prior to AS diagnosis, which is in line with the most former cases (1, 8, 19-22). In terms of familial history and parents' consanguinity, same as most prior cases, the current case had no affected relatives or consanguineous parents. Therefore, among mentioned factors, age, site, size, clinical presentation, as well as BCC and SCC history can be worthy in facilitating diagnosis despite gender, family history, and parents' consanguinity.

In terms of histopathologic features, studies revealed the significant finding of foamy cell alterations of the tumoral cells, which resembled a reactive xanthogranulomatous process. The most important differential diagnoses include xanthoma, dermatofibroma (clear cell variant), and sebaceous carcinoma (17, 25-27). Critical features for final diagnosis include irregularly shaped anastomosing vascular channels with highly infiltrative architecture and poor demarcation, as well as the multilayering of endothelial cells with nuclear atypia and increased mitoses (28-30). These changes are supported by immunohistochemical markers (30, 31). Compared to the previous reports of foamy cell appearance for AS, histopathologic studies of our case showed more mitoses, pleomorphism, and atypia (Table 2), and for the first time, foamy change were observed in the histopathologic examination of an XP patient with AS. A panel of immunohistochemical stains showed that the present tumor was positive for the Ki67 marker and the vascular markers including CD31 and CD34. However, the markers of Pan CK, MelanA, S100, HMB45, CK7, CEA, and CD68 were negative. It has been shown that both CD31 and CD34 are positive in almost all AS cases occurred in XP patients, which would be helpful in differentiation of AS form the mimics (Table 2). 

Regarding the treatment options for patients with AS, the traditional surgical excision procedure with or without postoperative radiation therapy remains the gold standard of care (32). Nonetheless, the role of adjuvant therapy is still a matter of debate, and a retrospective analysis of 764 cases indicated that only surgery, not radiotherapy or chemotherapy, is associated with improved survival (33). Even after the complete resection of the tumor, cutaneous AS has a significant risk of recurrence (32). Fujisawa* et al.* found that taxane maintenance therapy is effective for patients who had surgical resection and postoperative radiation and can lower the chance of recurrence (34). Recent investigations have focused on the functional features of AS, and some of the findings have led to the discovery of novel therapeutic targets. For instance, survivin was discovered to be a potential marker and therapeutic target for cutaneous ASs in a study (35). Systemic therapies are required for individuals with unresectable or metastatic tumors. Few clinical trials have evaluated systemic therapies for cutaneous AS. However, several studies have recommended paclitaxel as the first line of treatment, followed by pazopanib, bevacizumab, propranolol, and trabectedin, as the second-line treatment options (36-40).

Our patient was treated surgically with a forehead flap and showed no signs of recurrence or metastasis after 2 year follow-up. Most previous cases were also treated with surgical excision except for a few patients in the advanced disease stage who received chemotherapy and radiotherapy (1). Generally, the patients had no signs of recurrence. However, two 15 and 21-year-old females experienced recurrence within 2 and 11 months, respectively, which may be related to the more aggressive nature of malignancies in younger ages (20, 22).

In summary, this case is the first reported example of cutaneous AS with a rare variant of intratumoral foamy cell change in a patient diagnosed with XP. The patient underwent surgical excision, which was an effective cure. The authors of this paper have worked on skin cancers and associated disorders, which could be useful and practical for other researchers (41-43). 

## Conclusion

Cutaneous ASs are rare presentation of XP patients and are usually found as an erythematous and small plaque or nodule on the head and neck of younger adult XP patients that often have a history of BCC and SCC. Foamy cell AS is a rare histopathological variant of AS that is difficult to diagnose, and immunohistochemical assessments are helpful in correct diagnosis. Awareness of such rare clinical and histopathological presentations in XP patients can help clinicians better diagnose and treat.

## Conflict of Interest

The authors declare no conflict of interest.

## Author Contributions

FM reported the histopathologic and immunohistochemical findings of the case. FM and AG designed the study. FM, AZ, AD and AM wrote the paper. AG edited the manuscript. All authors have read and approved the content of the manuscript.

## Financial Support

None.

## Ethics Committee Approval

Due to the research protocol at the Iran University of medical sciences, the ethical committee's approval for case reports is not needed; however, the patient's consent for publication was obtained.

## Informed Consent

Informed consent was obtained from the patient for publication, and the subjects' rights were protected.
